# Classifying shoulder implants in X-ray images using deep learning

**DOI:** 10.1016/j.csbj.2020.04.005

**Published:** 2020-04-15

**Authors:** Gregor Urban, Saman Porhemmat, Maya Stark, Brian Feeley, Kazunori Okada, Pierre Baldi

**Affiliations:** a University of California, Irvine School of Information and Computer Sciences, Irvine, CA, USA; b San Francisco State University, Computer Science Department, San Francisco, CA, USA; c University of California, San Francisco, Department of Orthopaedic Surgery, San Francisco, CA, USA

**Keywords:** Deep learning, Computer vision, Orthopedics, X-ray imaging, Total shoulder arthroplasty

## Abstract

Total Shoulder Arthroplasty (TSA) is a type of surgery in which the damaged ball of the shoulder is replaced with a prosthesis. Many years later, this prosthesis may be in need of servicing or replacement. In some situations, such as when the patient has changed his country of residence, the model and the manufacturer of the prosthesis may be unknown to the patient and primary doctor. Correct identification of the implant’s model prior to surgery is required for selecting the correct equipment and procedure. We present a novel way to automatically classify shoulder implants in X-ray images. We employ deep learning models and compare their performance to alternative classifiers, such as random forests and gradient boosting. We find that deep convolutional neural networks outperform other classifiers significantly if and only if out-of-domain data such as ImageNet is used to pre-train the models. In a data set containing X-ray images of shoulder implants from 4 manufacturers and 16 different models, deep learning is able to identify the correct manufacturer with an accuracy of approximately 80% in 10-fold cross validation, while other classifiers achieve an accuracy of 56% or less. We believe that this approach will be a useful tool in clinical practice, and is likely applicable to other kinds of prostheses.

## Graphical abstract

**Figure:**
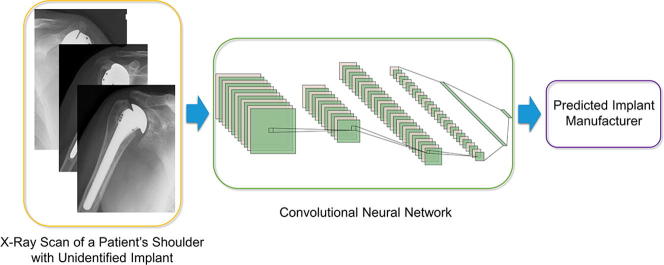

## 1 Introduction

Total Shoulder Arthroplasty (TSA) [1] is a common invasive procedure for treating damaged shoulder joints, where the shoulder ball is replaced with a prosthesis. The procedure is preceded and followed by a series of X-ray images to assess placement and fit.

Common reasons for undergoing TSA surgery are critical shoulder injuries or severe arthritis. The procedure mitigates pain and restores motion to the patients shoulder. There are several different manufacturers producing prostheses, and each of them offers several different models to better fit any type of situation and patient.

The prosthesis might – some or many years after it was implanted – come in need of repair or replacement. In some of these cases, the manufacturer and the model of the prosthesis may be unknown to the patients and their primary care doctors, for example when the surgery was conducted in another country where the patient has currently no access to the records. Another possible case of not knowing the exact manufacturer and model could be due ambiguity in medical records or medical images. At the present time, the task of identifying a prosthesis model in such cases is on the basis of rigorous examinations and visual comparisons of X-ray images taken from the implant by medical experts. This can be a monotonous task and requires time and effort for every new patient.

Detecting shoulder implants in X-ray images is not a well-studied problem, despite great advances in computer vision in recent years, predominantly made by deep Convolutional Neural Networks (CNNs). Our goal is to thoroughly evaluate the use of deep learning for classifying shoulder implants by manufacturer and compare it to more traditional classification methods. More precisely, we test custom models as well as five well-known deep convolutional neural networks with weights that were pre-trained on the large ImageNet data set [2]: VGG-16, VGG-19 [3], ResNet-50, ResNet-152 [4], DenseNet [5], and NASNet [6]. The use of pre-trained CNNs has been shown to be very successful in the context of X-ray data [7], [8], as well as for medical imaging data in other contexts [9], [10], [11], [12]. However, in some cases pre-training has actually been shown to be detrimental to model accuracy in biomedical image analysis [13].

The problem of identifying shoulder prostheses via X-ray images has not been studied before. Therefore, we evaluate a variety of more “traditional” classifiers besides deep learning models, such as Logistic Regression with SAGA (extension of Stochastic Average Gradient) [14], Random Forests [15], Gradient Boosting [16], and K-nearest Neighbors [17] to establish a more thorough baseline.

We focus on classifying shoulder implants by manufacturer only, instead of by model, due to insufficient amounts of images for each model. Nevertheless, the proposed model should be able to classify shoulder implants by both manufacturer and model once more data is collected.

## 2 Related work

To the best of our knowledge, no prior work exists on classifying shoulder implants, the closest being [18], where the authors propose a detection and segmentation algorithm for shoulder implants in X-ray images, based on the Hough Transform [19] for finding circles. However, they do not attempt classification. In [20] an approach to segment knee implants in X-ray images using template matching is proposed. Their algorithm uses various image processing techniques such as image smoothing, noise cancellation, sharpening, and Gaussian filtering, followed by template matching, but the authors acknowledge that the method is susceptible to noise and did not assess how well their method works quantitatively. Similarly, in [21] the authors identify knee prosthesis models in X-ray images using template matching and are reporting accuracies of 70% to 90%. However, their approach requires 3D CAD models of the implants to generate the templates and they could obtain only a single such implant model to evaluate their method. It would be difficult if not impossible to apply their method to our case of 16 different implant models. Other challenges for template matching are image artifacts, noise, variations in the way the image is captured, changes in image contrast, or variations in angles of image capturing. Deep Learning may prove to be more robust and more practical as only ordinary X-ray scans are needed for training and evaluation.

In [22] a classification system is proposed, which utilizes ensemble learning to detect fractures in human bone X-ray images with the main focus being on identifying fractures in long bones using K-Nearest Neighbors [17], SVM (Support Vector Machine) [23], and fully-connected neural networks. However, convolutional neural networks were neither used nor mentioned. A more recent study [24] utilizes deep convolutional neural networks to improve fracture detection in X-ray images taken from a variety of body parts.

## 3 Materials and Methods

### 3.1 Deep learning models

We use seven different convolutional architectures in total, six of which are well-known published architectures that are pre-trained on the ImageNet data set [2] and then fine-tuned on the shoulder X-ray image data set. For all pre-trained models, we discard their fully connected layers, as they are very likely to be specialized to the ImageNet data set and confer little benefit to our task, and insert one smaller fully-connected layer with random initial weights before re-training the model on the X-ray data.

#### 3.1.1 Pre-trained CNN

The pre-trained models that we use are (in order of publication date):

• The VGG-16 and VGG-19 networks introduced by [3] have 16 and 19 layers respectively. They have become well established for transfer learning tasks.
• Another (former) state-of-the-art CNN model is the deep residual network proposed by He et al. [4], of which we use the ResNet-50 and ResNet-152 variants. The main difference to non-residual networks such as VGG-16 is the use of (additive) skip connections.
• The DenseNet architecture [5] is inspired by residual networks. The main difference to ResNets is that each group of convolutional layers operates on the concatenated input from all previous groups of layers, by means of skip connections from and to all groups of layers.
• Motivated by Neural Architecture Search (NAS) framework [25], the dimensions of blocks of layers in the NASNet model [6] are optimized using reinforcement learning.

#### 3.1.2 Non-pre-trained CNN

**Fig. 1:**
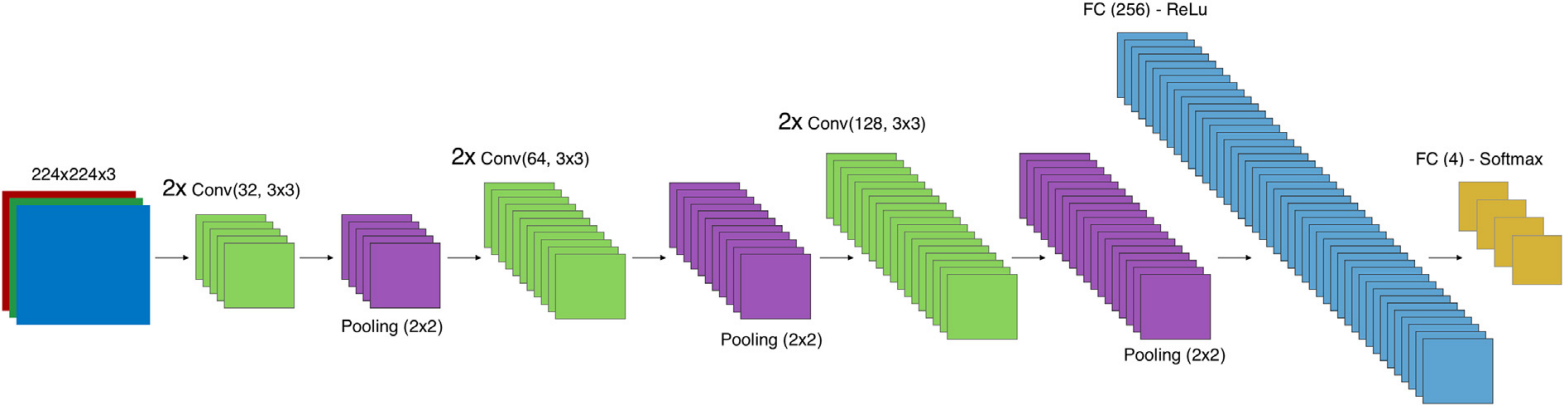
Architecture of the custom CNN model. Convolutional layers are denoted as *Conv*, max pooling layers as *Pool*, and fully connected layers as *FC*.

We build and train a custom CNN as a reference for not pre-training on external data. The model uses six convolutional layers, three max pooling layers, and one fully connected hidden layer. The architecture of this model is shown in Fig. 1.

• Conv(f,k): convolution layer with *f* convolutional filters of size *k*.
• Pool(k): max pooling layer with pooling size and stride *k*.
• FC(x): fully connected layer with *x* neurons.

We use rectified linear units in all layers but the output layer, which uses the Softmax function. We tested using batch normalization [26] and dropout [27], [28] as a means of regularization, but these did not improve the model performance.

### 3.2 Data set

**Fig. 2:**
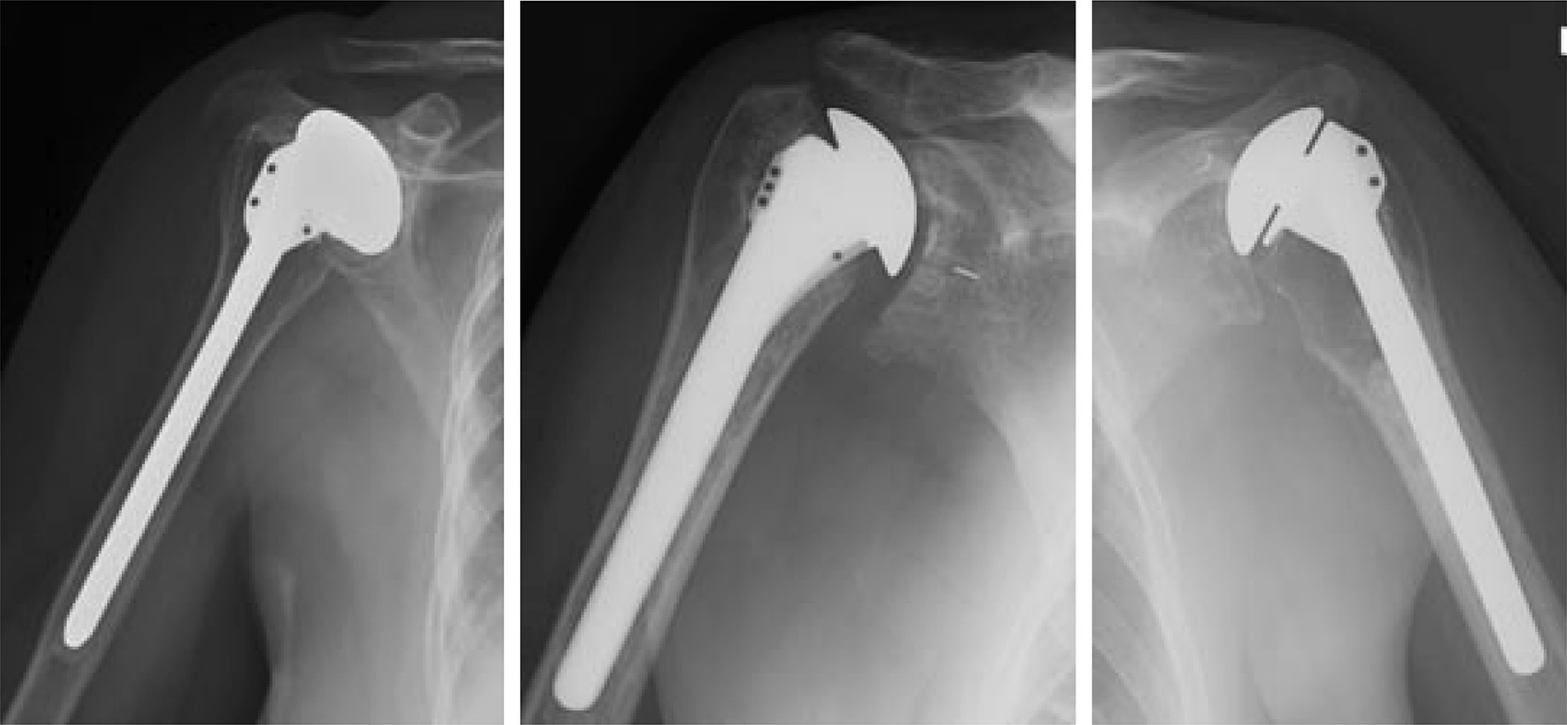
Examples of the data set: shoulder implants of three different manufacturers. Left to right: Cofield, Depuy, Zimmer.

The data set consists of 597 de-identified X-ray scans of implanted shoulder prostheses of four manufacturers and a total of 16 different models. Some of the images were obtained from the shoulder website of the University of Washington [29], and others from individual surgeons and manufacturers. All images that appeared to have been taken from the same patient were removed, which was the case for 8 out of an original set of 605 X-ray images. The final 597 samples in the data set contain 83 X-rays scans of implants from the manufacturer Cofield, 294 from Depuy, 71 from Tornier, and 149 are scans of implants made by Zimmer. Fig. 2 shows representative samples from the data set.

One of several challenges imposed by the data set is the variable and relatively low image resolution – the longest dimension of most of the images does not exceed 250 pixels and aspect ratios of the images differ. Other challenges are the variable and sometimes very low image contrast and class imbalance – a naive model predicting the most frequent manufacturer for all images would have an accuracy of 49.2%. The class imbalance problem would be far more severe if attempting to classify by model.

### 3.3 Pre-processing

In order to address the variable resolution of the images, we insert black borders such that all images are equally-sized squares – an alternative would be to rescale and interpolate images to a fixed size but this would introduce image distortion. We experimented with normalizing and enhancing the contrast of all images via histogram normalization. While it visually improved image quality, we found no improvement in model accuracy. We therefore opt for the simple and standard approach of normalizing images by subtracting their mean and dividing by their standard deviation.

### 3.4 Training and evaluation

We use data augmentation for training all models, including non-deep learning algorithms. Data augmentation is a common technique to improve the generalization of trained models [30], [31], essentially by increasing the effective amount of available labeled data. We apply random shifting, zooming, rotations, and random flipping of images. We use hyper-parameter optimization to find ideal parameters for the aforementioned operations: minimum and maximum number of pixels shifted and zoomed, and range of rotation angles. We use either Stochastic Gradient Descent (SGD) [32] or Adam [33] to train the CNN models, whichever works best for a given model, along with exponential decay of the learning rate during training.

We perform hyper-parameter optimization for every model using a fixed training/validation data split. We optimize the initial learning rate, rate of learning rate reduction, number of units in the final hidden layer, batch size for training, optimization algorithm (either Stochastic Gradient Descent (SGD) or Adam), and three parameters controlling the data augmentation: maximum range of random image rotations, range of image pixel shifts, and maximum amount of image stretching/zooming. To produce the results presented in Section 4 we take the best hyper-parameters found for any given model and train and evaluate it using stratified 10-fold cross validation, i.e. for each train/validation split of the data we use the same ratio of images per manufacturer as is present in the entire data set. We similarly optimize the hyperparameters of non-deep learning based algorithms.

We also experiment with augmenting test images of each split 20 times and average the model predictions across these augmentations to hopefully increase model accuracy. The approach of augmenting images at test-time is used in some ImageNet models, see e.g. [3], [34]. We re-use the data augmentation hyperparameters settings that were optimal for training.

Since the problem of classification of shoulder implants has not been studied before, we train several non-deep learning models as baseline, using Scikit-learn [35]. We use: (1) a Random Forest classifier with the Entropy split criterion using 500 trees in the forest; (2) multinomial Logistic Regression with L2 regularization optimized using SAGA; (3) Gradient Boosting with a learning rate of 0.15 and 15 estimators; and (4) a K-Nearest Neighbors classifier that uses the Euclidean distance metric with the value of K set to 35.

## 4 Results

**Fig. 3:**
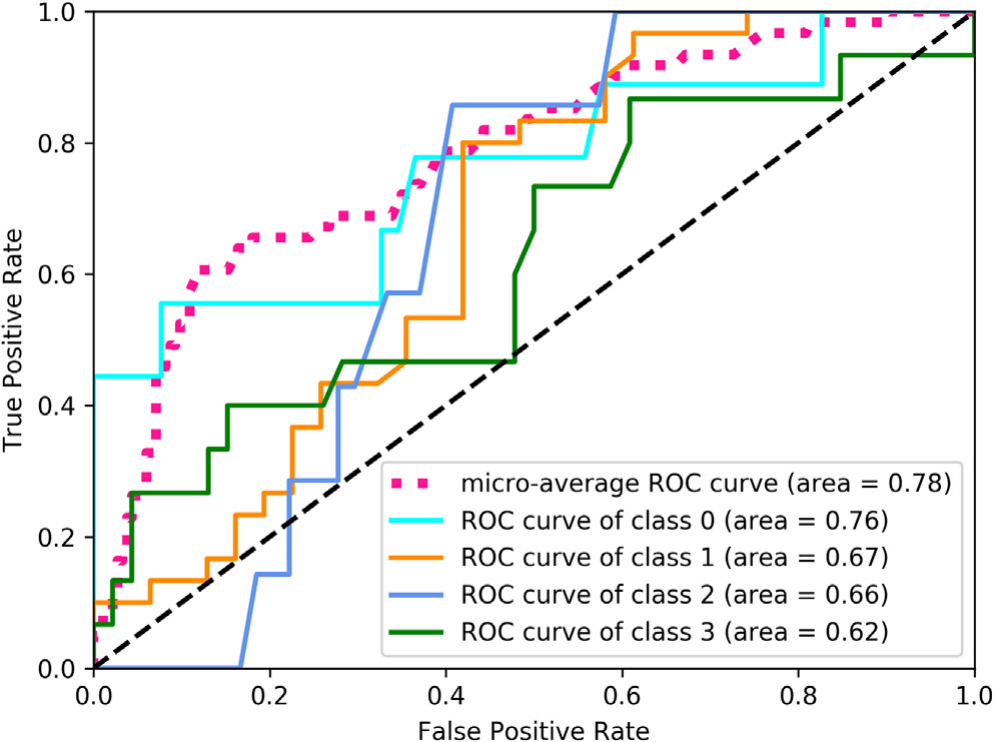
Receiver Operating Characteristic (ROC) curve for the Random Forest.

**Fig. 4:**
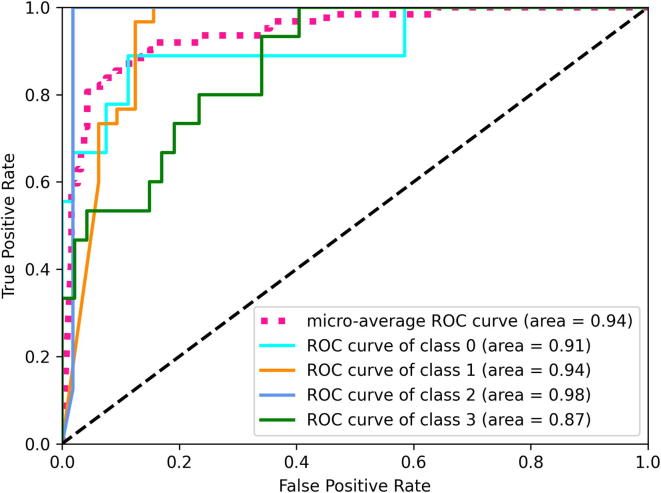
Receiver Operating Characteristic (ROC) curve for NASNet.

Table 1, Table 2, Table 3 present results obtained for different classifiers via 10-fold cross-validation as described in Section 3.4. Table 3 illustrates the performance of the CNN models with no pre-training on the ImageNet data set [2]. Fig. 3, Fig. 4 show the multi-class generalization of ROC (Receiver Operating Characteristic) plots for the best CNN and non-CNN model. Since ROC and AUC are defined only for binary classification problems, we follow [36] to compute the ROC/AUC one-versus-rest entities for every class and combine the different values into a single AUC value via micro-averaging, as this accounts for class-imbalance.

**Table 1:** Performance measures for non-deep learning classifiers. Shown are averages across 10-fold cross-validation, and standard deviation of the mean in parentheses. All methods were trained using data augmentation.

Classifier	Accuracy [%]	Precision	Recall	F1-Score	AUC
Random Forest	56 (1.)	0.62 (.03)	0.36 (.02)	0.51 (.03)	0.78 (.01)
Logistic Regression	53 (1.)	0.44 (.05)	0.31 (.01)	0.41 (.03)	0.73 (.01)
Gradient Boosting	55 (1.)	0.58 (.04)	0.34 (.01)	0.48 (.02)	0.75 (.01)
KNN	52 (1.)	0.49 (.04)	0.31 (.01)	0.43 (.02)	0.73 (.01)

**Table 2:** Performance measures for convolutional neural networks **with pre-training on ImageNet**. All models are *trained* with data augmentation, but we evaluated them both with and without test-time data augmentation. Shown are averages across 10-fold cross-validation and standard deviation of the mean in parentheses.

Classifier	Accuracy [%]	Precision	Recall	F1-Score	AUC
*No Test Data Augmentation*					
VGG-16	74.0 (2.3)	0.72 (.03)	0.68 (.02)	0.69 (.03)	0.93 (.01)
VGG-19	76.2 (1.6)	0.75 (.03)	0.69 (.03)	0.70 (.03)	0.93 (.01)
ResNet-50	75.4 (1.5)	0.75 (.02)	0.70 (.02)	0.71 (.02)	0.93 (.01)
ResNet-152	75.6 (2.0)	0.73 (.03)	0.69 (.02)	0.70 (.03)	0.92 (.01)
NASNet	80.4 (.8)	0.80 (.01)	0.75 (.02)	0.76 (.02)	0.94 (.00)
DenseNet-201	79.6 (.9)	0.79 (.01)	0.74 (.02)	0.74 (.01)	0.94 (.01)
					
*With Test Data Augmentation*					
VGG-16	75.2 (1.7)	0.74 (.02)	0.67 (.03)	0.68 (.03)	0.93 (.01)
VGG-19	76.2 (1.9)	0.75 (.03)	0.68 (.02)	0.69 (.03)	0.93 (.01)
ResNet-50	75.2 (1.8)	0.77 (.02)	0.67 (.03)	0.70 (.02)	0.92 (.01)
ResNet-152	74.5 (1.4)	0.71 (.03)	0.69 (.03)	0.69 (.03)	0.91 (.00)
NASNet	78.8 (1.8)	0.78 (.02)	0.73 (.03)	0.73 (.03)	0.93 (.01)
DenseNet-201	78.9 (2.0)	0.79 (.03)	0.74 (.03)	0.76 (.03)	0.93 (.01)

**Table 3:** Performance measures for convolutional neural networks **without pre-training**. Shown are averages across 10-fold cross-validation and standard deviation of the mean in parentheses.

Classifier	Accuracy [%]	Precision	Recall	F1-Score	AUC
VGG-16	55.6 (1.7)	0.46 (.02)	0.42 (.02)	0.42 (.02)	0.78 (.01)
VGG-19	57.0 (1.6)	0.50 (.03)	0.43 (.02)	0.43 (.02)	0.78 (.01)
ResNet-50	53.8 (1.7)	0.39 (.06)	0.34 (.03)	0.31 (.04)	0.74 (.02)
ResNet-152	53.4 (1.2)	0.38 (.03)	0.36 (.02)	0.34 (.02)	0.77 (.01)
NASNet	51.8 (1.5)	0.22 (.04)	0.29 (.02)	0.23 (.03)	0.71 (.02)
DenseNet-201	54.0 (1.3)	0.46 (.02)	0.40 (.02)	0.39 (.02)	0.79 (.01)
Custom CNN	56.0 (1.4)	0.42 (.02)	0.42 (.02)	0.41 (.02)	0.78 (.01)

Random Forests are the best performing non-deep-learning classifier and reach an accuracy of 56% (see Table 1) when using data augmentation during training, which is slightly better than the chance level of 49.2% for guessing the majority class. The custom convolutional neural network without pre-training on external data (Table 3, bottom) reaches the same accuracy. On the other hand, all models that were pre-trained on the ImageNet data set perform significantly better, with accuracy values ranging from 74% to 80% (see Table 2). This difference is statistically significant for all models, even at a very strict p-value of 0.001 of the two-tailed student-t test. For this test and p-value and with 18 degrees of freedom the critical value is 3.922. For example for the NASNet model we have t=(80.4-51.8)/2=14.3 which fulfills the t>3.922 criterion by a wide margin.

While it is not surprising that pre-trained models would perform better, the difference is considerable. All non-pretrained models seem to overfit a lot on this data, which is especially true for the ImageNet models when trained starting from scratch (cf. Table 3), as all these models have many parameters. We suspect that some of the factors that make classification hard are: (1) a large intra-class variability, as manufacturer offers multiple models; (2) a low inter-class variability, as all implants look roughly alike and no trivial features (such as color or context) exist that would help in distinguishing them; (3) the high variability in image size, quality, and device used to generate it; and (4) class and sub-class imbalance in the data, i.e. the number of images per manufacturer as well as per model differ.

As can be seen in Table 2 all pre-trained models reach relatively comparable levels of performance, and are all significantly better in all metrics compared to models without pre-training (see Table 3). On the other hand, using test-time data augmentation with model prediction averaging seems to not have any significant impact on model performance – in some metrics it performs slightly better, in others worse. A possible reason is that the hyperparameters were set to values that are too extreme – we re-used the optimal settings from the training phase as we didn’t want to further optimize them and risk over-fitting on the small data set.

Furthermore, we test how well the features learned by pre-trained CNNs on ImageNet transfer to the implant classification task when not fine-tuned on the X-ray data. For this we run the pre-trained VGG-16 and −19 models on the X-ray data set and collect the activations of their final pooling layers, thus omitting the hidden layers that are more ImageNet data specific. We repeat this step ten times on differently augmented version of the images as a means of data augmentation. Subsequently, we train a multilayer perceptron (MLP) classifier on these features using the same ten-fold cross validation procedure as done in all other experiments, making sure to keep all features belonging to the same image in either only the train or test splits and not mix them. The results, shown in Table 4, are significantly better than all non-pretrained models in Table 1, Table 3, showing that the features learned on external data are extremely helpful even though those were not medical images. However, it is also clear when comparing Table 2 to Table 4 that fine-tuning the entire CNN is better than just fine-tuning the top hidden layers.

**Table 4:** Performance of MLP classifiers trained on features extracted from pre-trained ImageNet CNNs. Shown are averages across 10-fold cross-validation, and standard deviation of the mean in parentheses. Trained using data augmentation.

Features	Accuracy [%]	Precision	Recall	F1-Score	AUC
VGG-16	72.3 (1.)	0.77 (.01)	0.61 (.02)	0.65 (.02)	0.90 (.01)
VGG-19	72.2 (2.)	0.78 (.02)	0.64 (.03)	0.67 (.03)	0.91 (.01)

In a final experiment we assess the effect of using data augmentation during training (see Table 5). As anticipated, training with data augmentation has a large positive effect on model performance: the best CNN in terms of accuracy (NASNet) is able to reach an accuracy of 80.4% when trained with data augmentation, but merely 64.5% when trained without. A similar drop in performance is observable in all metrics recorded.

**Table 5:** Performance measures for convolutional neural networks **without using any data augmentation**. Shown are averages across 10-fold cross-validation and standard deviation of the mean in parentheses.

Classifier	Accuracy [%]	Precision	Recall	F1-Score	AUC
VGG-16	58.7 (2.5)	0.54 (.03)	0.45 (.03)	0.45 (.04)	0.81 (.02)
VGG-19	63.6 (1.6)	0.61 (.02)	0.53 (.03)	0.54 (.03)	0.84 (.01)
ResNet-50	59.6 (2.2)	0.56 (.02)	0.49 (.02)	0.49 (.02)	0.83 (.01)
ResNet-152	59.5 (1.2)	0.54 (.03)	0.47 (.02)	0.48 (.02)	0.83 (.01)
NASNet	64.5 (3.4)	0.62 (.05)	0.52 (.04)	0.54 (.04)	0.85 (.02)
DenseNet-201	65.9 (2.4)	0.65 (.03)	0.55 (.03)	0.57 (.03)	0.86 (.02)
Custom CNN	50.8 (2.4)	0.39 (.04)	0.32 (.01)	0.30 (.02)	0.73 (.01)

## 5 Discussion

Certain elements deserve additional consideration, that become relevant when extending or deploying the presented work.

• Class imbalance: If we assumed that the current data set’s implant manufacturer ratio was representative of the true prevalence of implants in a typical patient, then training on the entire data set and using the resulting model “as-is” would be optimal, as the model’s bias would match the actual prevalence. But if the true prevalence was different, one would have to either dynamically over- or undersample certain manufacturer models during training, or re-balance the model output confidence. It should be noted that dealing with imbalanced data is still an open problem [37], so there is no solution that is guaranteed to be optimal.
• It is also worthwhile to consider the case that a test image could come from a manufacturer not contained in the training set. One way to address this is to assess the model output confidence scores for the different classes and check if their distribution fulfills certain criteria. Alternative methods have been proposed in recent work such as [38], which promises to do better than simply using the existing model outputs.
• A natural way to extend this work could be to classify shoulder implants by both manufacturer and model, and to include additional manufacturers. In either case this requires gathering more data to train models with acceptable accuracy.

## 6 Conclusions

We evaluate the use of deep learning for classifying shoulder implants in X-ray images by manufacturer and compare it with a baseline of other classifiers. Out of seven deep learning architectures tested, we find that all well-known ImageNet models perform well, with NASNet [25] taking the lead with an accuracy of 80.4%. We find that pre-training the CNNs on a different large computer vision data set such as ImageNet [2] is crucial to obtain good results, and that fine-tuning the entire CNN model on the task-specific X-ray data set is better than only fine-tuning the top hidden layers. We compare the performance of the neural networks with other classifiers, including Gradient Boosting, Random Forests, Logistic Regression, and K-nearest Neighbors. Ultimately, we find that pre-trained and then fine-tuned CNNs outperform all other classifiers and all non-pre-trained CNNs by a significant margin, with accuracies of pre-trained CNNs reaching a range of 74% to 80% compared to accuracies of merely 51% to 56% for all classifiers without pre-training (including CNNs and non-deep learning algorithms). We also examined the effectiveness of data augmentation, and found it to be crucial, as training even pre-trained CNNs without data augmentation on the X-ray data set leads to accuracies of only 59% to 66%, constituting a significant drop by approximately 14 percentage points across all models.

## Funding

This material is based upon work supported by the National Science Foundation under award number 1633631 and 1839429.

## Declaration of Competing Interest

The authors declare that they have no known competing financial interests or personal relationships that could have appeared to influence the work reported in this paper.
